# COLEC12 regulates apoptosis of osteosarcoma through Toll‐like receptor 4–activated inflammation

**DOI:** 10.1002/jcla.23469

**Published:** 2020-08-21

**Authors:** Guang‐Zhang Li, Jian‐Feng Deng, Ying‐Zhao Qi, Ran Liu, Zhi‐Xin Liu

**Affiliations:** ^1^ Department of Orthopedics the First Hospital of Qinhuangdao Hebei China

**Keywords:** COLEC12, inflammation, osteosarcoma, TLR4

## Abstract

**Objective:**

To investigate the role of COLEC12 in osteosarcoma and observe the relationship between COLEC12 knockdown and the inflammation of osteosarcoma. Then, further explore whether the process is regulated by TLR4.

**Method:**

GEPIA and TCGA systems were used to predict the potential function of COLEC12. Western blot and RT‐PCR were used to analyze the protein expression, or mRNA level, of COLEC12 in different tissue or cell lines. The occurrence and development of osteosarcoma were observed by using COLEC12 knockdown lentivirus. The inflammation indexes of osteosarcoma, in vitro and in vivo, were explored. TLR4 knockdown lentivirus was applied to the relationship between COLEC12 and TLR4.

**Results:**

COLEC12 expression in SARC tumor tissue was higher than in normal, and a high expression of COLEC12 in SARC patients had a worse prognostic outcome. Pairwise gene correlation analysis revealed a potential relationship between COLEC12 and TLR4. The COLEC12 expression and mRNA level in the tumor or Saos‐2 cells were increased. COLEC12 knockdown lentivirus could inhibit osteosarcoma development, in vivo and vitro, through reducing tumor volume and weight, weakening tumor proliferation, migration, and invasion, and enhancing apoptosis. Furthermore, COLEC12 knockdown could increase inflammation of osteosarcoma, in vivo and in vitro, through inducing myeloperoxidase (MPO), TLR4, NF‐κB, and C3, and expression of related inflammatory factors. Finally, TLR4 knockdown lentivirus inhibits the progress of inflammation after COLEC12 regulation, in vivo and vitro.

**Conclusion:**

COLEC12 may be able to regulate apoptosis and inflammation of osteosarcoma, and TLR4 may be the downstream target factor of COLEC12 in inflammation.

## Graphical abstract

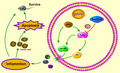

## 1 INTRODUCTION

Osteosarcoma (OS) is a tumor characterized by the presence of malignant mesenchymal cells produced in the bone stroma, and one of the most malignant bone tumors with high rate of morbidity and mortality in children and young adults.^1^, ^2^ The traditional prognostic parameters have been inaccuracy and inadequacy in daily clinical work.^3^ Accordingly, finding novel prognostic parameters is warranted to help improve the survival of osteosarcoma patients.

The tumor microenvironment exerts different effects on virgin mesenchymal stem cells (MSCs), including recruiting cytokines, stimulating by the paracrine network, and undergoing a series of functional transformations.^4^, ^5^

Immune surveillance is a complex biological process that combines the recognition of tumor cells and specific effector cells tightly controlled by regulatory immune cells. In turn, tumor cells can secrete soluble factors to down modulate the immune surface markers and dampen the immune system.^6^ Inflammation then plays a dual function by slowing down the tumor progression. Of the potential new targets, the environment of inflammation has received considerable attention and become an immunotherapy challenge for re‐inducing an antitumoral response.^7^, ^8^, ^9^

TNF‐α and ILs are pro‐inflammatory cytokines produced by lymphocytes and macrophages, which can induce apoptosis of tumor cells and are associated with the progression of several types of tumors, including OS.^10^, ^11^ In addition, TNF‐α and ILs are transcriptionally controlled by NF‐kB and C3 signaling^12^, ^13^; therefore, searching the upstream key factors to regulate the inflammatory response may be a vital step in the treatment of osteosarcoma.

COLEC12 (collectin subfamily member 12, CL‐12, or CL‐P1) is a pattern recognition molecule (PRM) of the innate immune system, which is expressed from the COLEC12 gene located on chromosome 18p11.32.^14^ CL‐12 has been suggested to be involved in leukocyte recruitment and cancer metastasis.^15^ It has also been found that increased levels of COLEC12 expression were seen in cancer‐associated stromal cells,^16^ which suggests that there is a correlation with tumor inflammation; however, the mechanism of COLEC12 in inflammation of osteosarcoma has not been fully clarified.

Toll‐like receptor 4 (TLR4) is a pivotal promoter of inflammation, which relates to NF‐kB and C3 signaling.^17^, ^18^ However, it has not yet been reported whether the role of COLEC12 for inflammation in osteosarcoma is regulated by TLR4.

In this study, we focused on the role of COLEC12 in osteosarcoma and COLEC12 knockdown lentivirus to boost inflammatory progress and tumor development, in vivo and in vitro. Furthermore, we also used TLR4 knockdown lentivirus to reduce TLR4 expression and inhibit the progress of inflammation after COLEC12 regulation. Our findings indicate that COLEC12 is possibly able to regulate inflammation in osteosarcoma when mediated through TLR4. These results may provide a new strategy to osteosarcoma development by inflammation.

## 2 MATERIALS AND METHODS

### 2.1 Cell lines and cell culture

hFOB is human osteoblast cell line, and 143B, MG‐63, Saos‐2, and HOS are human osteosarcoma cell lines. Cells were cultured in DMEM containing 10% FBS and supplemented with an antibiotic, non‐essential amino acid solution, and MEM vitamin mixture and cultured in an incubator maintained at 5% CO_2_ and 37°C, as described previously.^19^

### 2.2 Animals

2‐day or 4‐week‐old healthy male Balb/c mice were used and housed in a temperature‐ and light‐controlled environment under pathogen‐free conditions and provided unlimited access to food and water and 12/12‐hour light/dark, with humidity 60 ± 5% and temperature 22 ± 3°C. All animals used in this experiment were cared for in strict accordance with the Guide for the Care and Use of Laboratory Animals (NIH Publication No. 85‐23, Revised 1996).

### 2.3 Reagents and antibodies

The following reagents and antibodies were used in this study: hFOB cells (CL‐0353, Procell), 143B cells (CRL‐8303, ATCC), MG‐63 cells (CL‐0157, Procell), Saos‐2 cells (CL‐0202, Procell), and HOS cells (CL‐0360, Procell). Anti‐COLEC12 (ab81136; Abcam), anti‐BAX (ab32503; Abcam), anti‐BCL‐2 (ab182858; Abcam), anti‐cleaved caspase‐3 (ab2302; Abcam), anti‐cleaved PARP1 (ab2302; Abcam), anti‐MPO (ab9535; Abcam), anti‐TLR4 (ab13556; Abcam), anti‐NF‐κB (BM3940, Boster), anti‐IL‐1β (ab23437; Abcam), anti‐IL‐18 (ab71495; Abcam), anti‐TNF‐α (ab1793; Abcam), anti‐C3 (ab200999; Abcam), and anti‐β‐actin (M01263‐2, Boster), followed by secondary antibodies conjugated to horseradish peroxidase anti‐rabbit IgG (H + L) (AS014, ABclonal) and anti‐mouse IgG (H + L) (AS003, ABclonal).

### 2.4 COLEC12 knockdown and TLR4 lentivirus administration

COLEC12 knockdown via lentivirus (sh‐COLEC12), TLR4 knockdown via lentivirus (sh‐TLR4), and a control lentivirus were designed and chemically synthesized by GenePharma Corporation, in Shanghai, China. The lentivirus vectors were stored at −80°C, accordingly. Osteosarcoma cells were inoculated into 6‐well plates with 2 × 10^5^ cells/well, and the serum‐free medium was replaced after the density was higher than 70%. 10 μL of knockdown lentivirus was diluted in the medium and incubated for 6 hours. The cell protein or mRNA was collected after 72 hours. Cells transfected with lentivirus were used to establish an osteosarcoma mouse model after passage. The sequences of COLEC12 and TLR4 lentivirus are shown in Tables 1 and Table 2.

**Table 1:** Sequences of COLEC12

Name	Sequence
sh‐COLEC12‐301	5’‐TTCTATCATATTATTATACA‐3’
sh‐COLEC12‐485	5’‐AAAGCTATCAGCACCAACTC‐3’
sh‐COLEC12‐900	5’‐TCAAAATCTGCAGCAGGTTT‐3’
Negative control	5’‐CTTGCTAACAATCACAGTAG‐3’
Control‐shCOLEC12	5’‐ATGGAAACATCTCGCCAAAC‐3’

**Table 2:** Sequences of TLR4

Name	Sequence
sh‐TLR4‐362	5’‐CTGCATAGAGGTAGTTCCTA‐3’
sh‐TLR4‐543	5’‐GAAATTGAAACAATTGAAGA‐3’
sh‐TLR4‐1325	5’‐CCCTTTCTTAAAAGTTTGAC‐3’
Negative control	5’‐GAGCTTCAACCCCTTGAAGA‐3’
Control‐shTLR4	5’‐GTGGCTGGATTTATCCAGGT‐3’

### 2.5 Osteosarcoma mouse model

Four‐week‐old male Balb/c mice were fed in the specific pathogen–free (SPF) environment, and osteosarcoma cells were injected subcutaneously. After five days, vital signs and inoculation sites of the mice were observed. Experiments were divided into five groups: Con group, sh‐COLEC12 group, control sh‐COLEC12 group, sh‐TLR4 group, and control sh‐TLR4 group. Tumor tissue was collected after modeled for 20d, and tumor volume = 0.5 × length × width^2^ (mm^3^).

### 2.6 Western blot

The amounts of 40 μg of protein from each brain sample were subjected to 12% SDS‐polyacrylamide gel electrophoresis and transferred to a nitrocellulose membrane. All data were detected with the ChemiDoc^™^ Touch Imaging System and analyzed with the Image Lab 3.0 software (Bio‐Rad).

### 2.7 Quantitative RT‐PCR

Total RNA was extracted from frozen brain using Reagent Kit (TaKaRa Biotechnology). A total of 40 μL RNA was reverse‐transcribed into cDNA. Quantitative PCR was performed as described.^20^ Primer sequences for the amplification of COLEC12 and β‐actin. COLEC12 mRNA level was calculated by its ratio to β‐actin.

### 2.8 Immunofluorescence (IF) staining

The expression of MPO was detected by IF staining. Paraffin‐embedded tissue sections (5 mm thickness) were dewaxed in xylene, then dehydrated using graded concentrations of alcohol, and incubated with 3% H_2_O_2_ to inhibit endogenous peroxidase. After being blocked in 10% goat serum for 10 minutes at room temperature, they were incubated with the primary antibody (anti‐MPO 1:200, ab3683, Abcam) in a blocking solution overnight at 4°C. After slides were washed in PBS, HRP‐labeled anti‐rabbit IgG (1:100; AS014, ABclonal) was applied for 30 minutes at 37°C, and then washed with PBS again. Then, images were obtained with a Nikon EclipseNi inverted microscope (TE2000, Nikon).

### 2.9 TUNEL staining

Tissue fraction was determined by TUNEL assay. The TUNEL assay was performed according to the instruction manual. Images were photographed by fluorescent microscopy at 400 × magnification.

### 2.10 Flow cytometry assay (FCM)

The culture medium was placed in a centrifuge tube, washed with PBS, digested with trypsin, and blown off individual cells. The culture solution was collected and centrifuged with a 15‐ml centrifuge tube at 1000 rpm for 6 minutes, and the supernatant was discarded. 1ml pre‐cooled PBS was added, and the cells were resuspended and counted, and performed as described in reference.^21^

### 2.11 MTT

After stirring the solution for 30 minutes, the bacteria were removed with a microporous (diameter 0.22 m) filter and stored at 4°C away from light, after packaging. Cells in the logarithmic growth stage were inoculated into 96‐well plates in groups. Cells with a concentration of 200 mL of 4 × 10^4^/mL were inoculated into each well. Transfection was carried out 24 hours after incubation in a constant temperature incubator at 37°C and 5% CO_2_. The solution was added to the cells of each group in the sequence of time since transfection (20 μL/well). The cells were transferred to the incubator for further incubation for 6 hours, and MTT was reduced to formazan. After absorbing the medium, the crystals of formazan were dissolved by adding 150 μL DMSO and oscillating for 10 minutes. Blank control wells (no cells, just culture medium) were set, and the blank control wells were zeroed for the final colorimetric test. OD values of each hole were determined by enzyme‐linked immunoassay (490 nm).

### 2.12 Transwell migration and invasion assay

The Transwell chamber was pretreated with Matrigel and dried at 37°C for 1 hour. Other procedures were the same as for the Transwell migration assay. The results of the Transwell migration and invasion assay were also calculated according to the number of transferred cells.

### 2.13 Cell cycle analysis

AtT‐20 cells were plated in six‐well plates at a density of 2 × 10^5^ cells per well. After 12 hours, various concentrations (0, 25, 50, 100 mmol/L) of eriodyctiol were added to each well, and cells were incubated for an additional 48 hours. Detailed procedures were performed as described in reference.^22^

### 2.14 Statistical analysis

Data were all expressed as mean ± standard. Statistical analysis and correlation analysis were performed on the data using GraphPad Prism 6.0 (GraphPad Software). The survival analysis was performed using SPSS 19.0 (IBM). Differences were analyzed using one‐way ANOVA, and multiple comparisons were analyzed using the Sidak test. All differences were considered statistically significant at a *P* value < .05.

## 3 RESULTS

### 3.1 Bioinformatic analysis of COLEC12 in sarcoma by GEPIA and TCGA systems which to predict osteosarcoma

**Figure 1:**
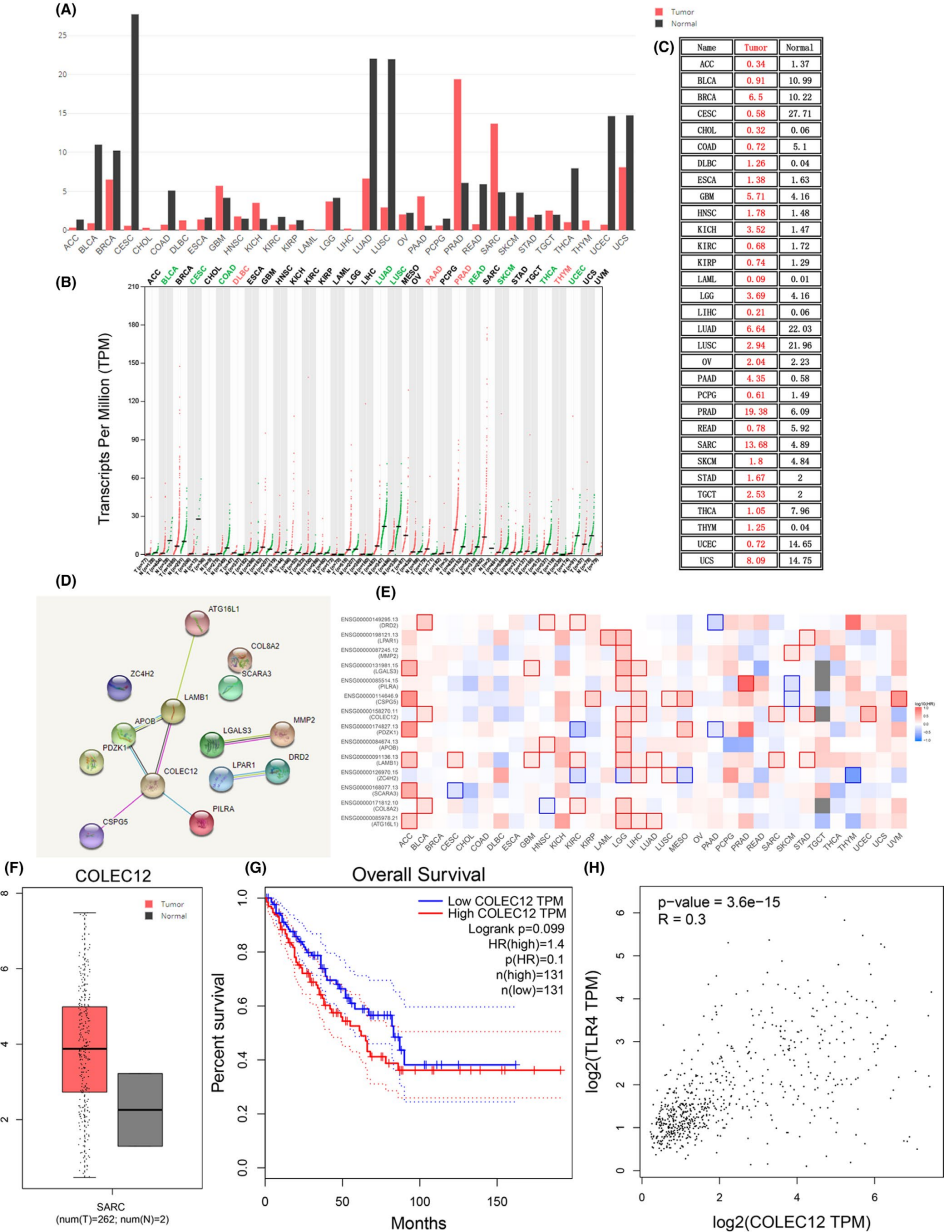
A, Expression profile, B, specific quantified value, and C, transcripts per million in different cancers by GEPIA and TCGA systems. D, Interacting proteins of string assay and E, survival map for COLEC12 and its interacting proteins in SARC. F, Isoform analysis in box plot and G, survival analyses in SARC cancer type. H, Pairwise gene correlation analysis of COLEC12 and TLR4

The predicted results of COLEC12 in sarcoma by GEPIA and TCGA systems are shown in Figure 1. As shown in Figure 1A‐C, the expression profile and transcripts per million were quantified in different cancers; COLEC12 expression in SARC tumor tissue was higher than the normal. The interacting proteins for COLEC12 by String assay were ATG16L1, LAMB1, APOB, PILRA, CSPG5, ZC4H2, PDZK1, COL8A2, SCARA3, LGALS3, MMP2, LPAR1, and DRD2 (Figure 1D). The survival map for COLEC12 and its interacting proteins is shown in Figure 1E, which had significant results in SARC of COLEC12. For the isoform analysis in box plot and survival analyses (Figure 1F‐G), we can easily conclude that COLEC12 isoform in SARC cancer type was overexpressed compared with the normal tissue. Meanwhile, given the high expression of COLEC12 isoform, the patients in SARC had a worse prognostic outcome. Pairwise gene correlation analysis of COLEC12 and TLR4 is shown in Figure 1H, which reveals a potential relationship between COLEC12 and TLR4.

### 3.2 The different level of COLEC12 expression at different tissue or cell line

**Figure 2:**
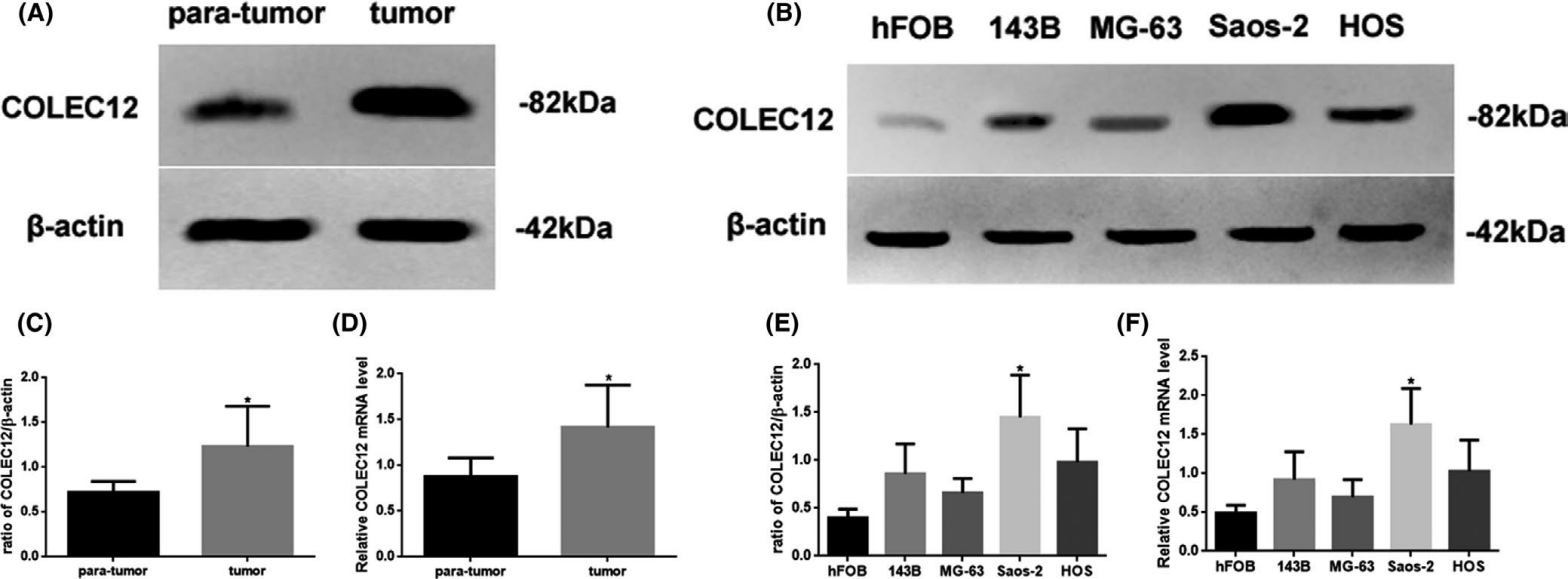
A, Western blot assay of COLEC12 expression in tumor and para‐tumor tissue. B, Western blot assay of COLEC12 expression in hFOB, 143B, MG‐63, Saos‐2, and HOS cells. C, Quantification. and D, Quantitative RT‐PCR assay for mRNA level of COLEC12 in different tissues. E, Quantification. and F, Quantitative RT‐PCR assay for mRNA level of COLEC12 in different cells. Protein and mRNA levels were normalized to β‐actin. (tumor or Saos‐2 cells vs. para‐tumor or hFOB cells, ^*^ *P* < .05, n = 6 per group; all data were represented as mean ± standard error)

The different level of COLEC12 expression at different tissue or cell line was examined by Western blot (Figure 2). The COLEC12 expression and mRNA level in an osteosarcoma tumor were increased (*P* < .05), compared with para‐carcinoma tissue (Figure 2A, C, and D). In addition, the COLEC12 expression and mRNA level in Saos‐2 cells were most significantly increased (*P* < .05), compared with hFOB (Figure 2B, E, and F). We used Saos‐2 as the osteosarcoma model for the follow‐up experiments.

### 3.3 *Knockdown COLEC12 could regulate osteosarcoma development* in vitro

**Figure 3:**
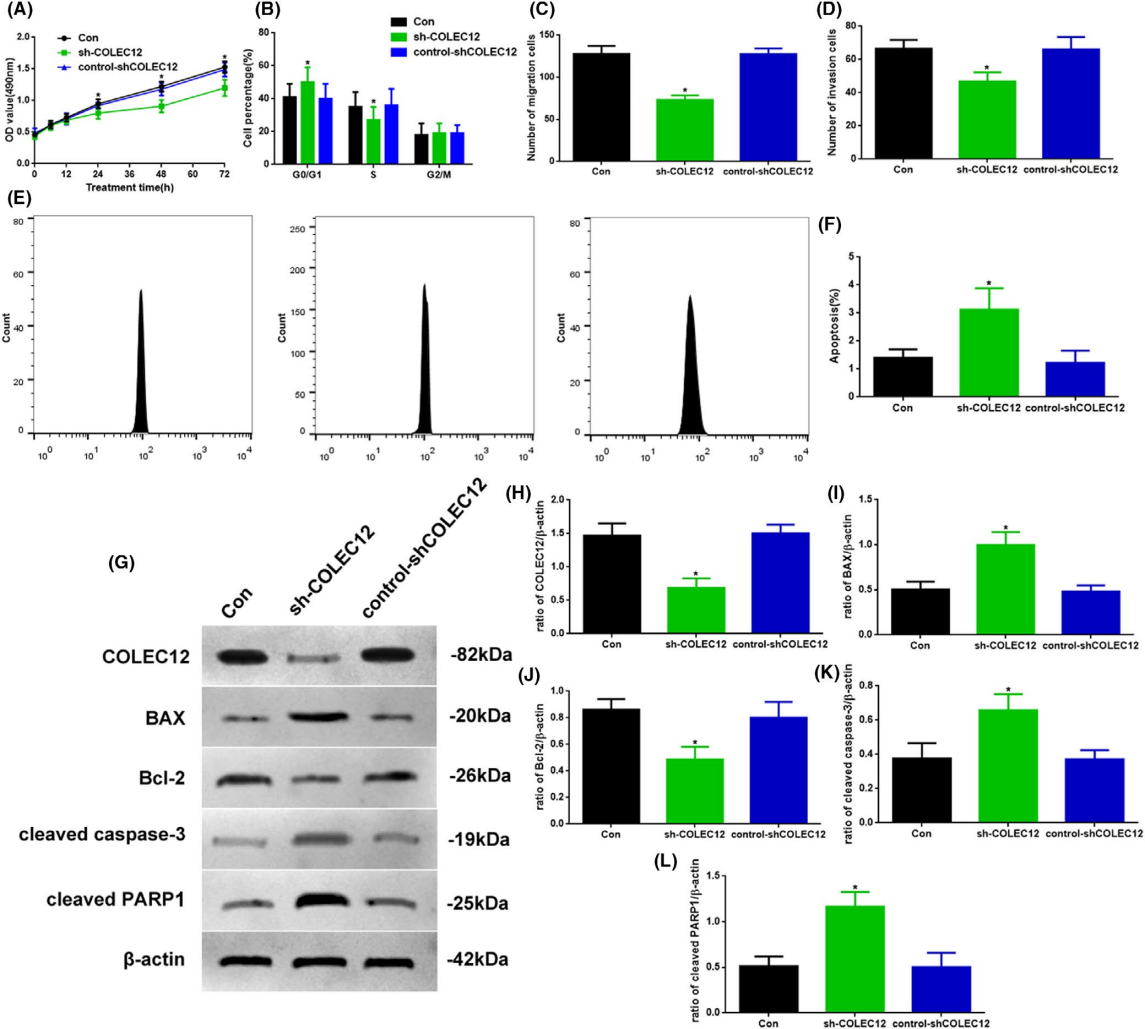
A, Proliferation assay in 72 hours, B, cell cycle assay, C, migration assay, and D, invasion assay at 72 hours of Con, sh‐COLEC12, and control‐shCOLEC12 groups. E, F, The flow cytometry assay of apoptosis. G, Western blot assay of COLEC12, BAX, Bcl‐2, cleaved caspase‐3, and cleaved PARP1 expression in vitro. Quantification of (H) COLEC12, (I) BAX, (J) Bcl‐2, (K) cleaved caspase‐3, and L, cleaved PARP1. Protein levels were normalized to β‐actin. (sh‐COLEC12 vs. other groups, ^*^ *P* < .05, n = 6 per group)

As COLEC12 expression in the tumor was increased, probably correlated with tumor progression, we attempted a method for decreasing the COLEC12 level: COLEC12 knockdown lentivirus in vitro (Figure 3). We tested the proliferation, cell cycle, migration, and invasion of Con, sh‐COLEC12, and control‐shCOLEC12 Saos‐2 cells. The OD value in 24, 48, and 72 hours tested by MTT in the sh‐COLEC12 group was significantly lower than in the other two groups (Figure 3A). The distribution in G0/G1 phase in sh‐COLEC12 cells was significantly higher than in the other two groups and in the contrary results in the S phase (Figure 3B, *P* < .05). The migration and invasion ability test by transwell assay in the sh‐COLEC12 group were also decreased (Figure 3C‐D, *P* < .05). To investigate the function of apoptosis by COLEC12, we used flow cytometry to observe the changes (Figure 3E), and the apoptotic level in the sh‐COLEC12 group was increased (Figure 3F, *P* < .05). In addition, we also detected related proteins of apoptosis by Western blot (Figure 3G). The expression of BAX, cleaved caspase‐3, and cleaved PARP1 in the sh‐COLEC12 group was increased (*P* < .05), compared with other groups; COLEC12 and Bcl‐2 showed contrary results (Figure 3H‐L).

### 3.4 *Knockdown COLEC12 could regulate osteosarcoma development* in vivo

**Figure 4:**
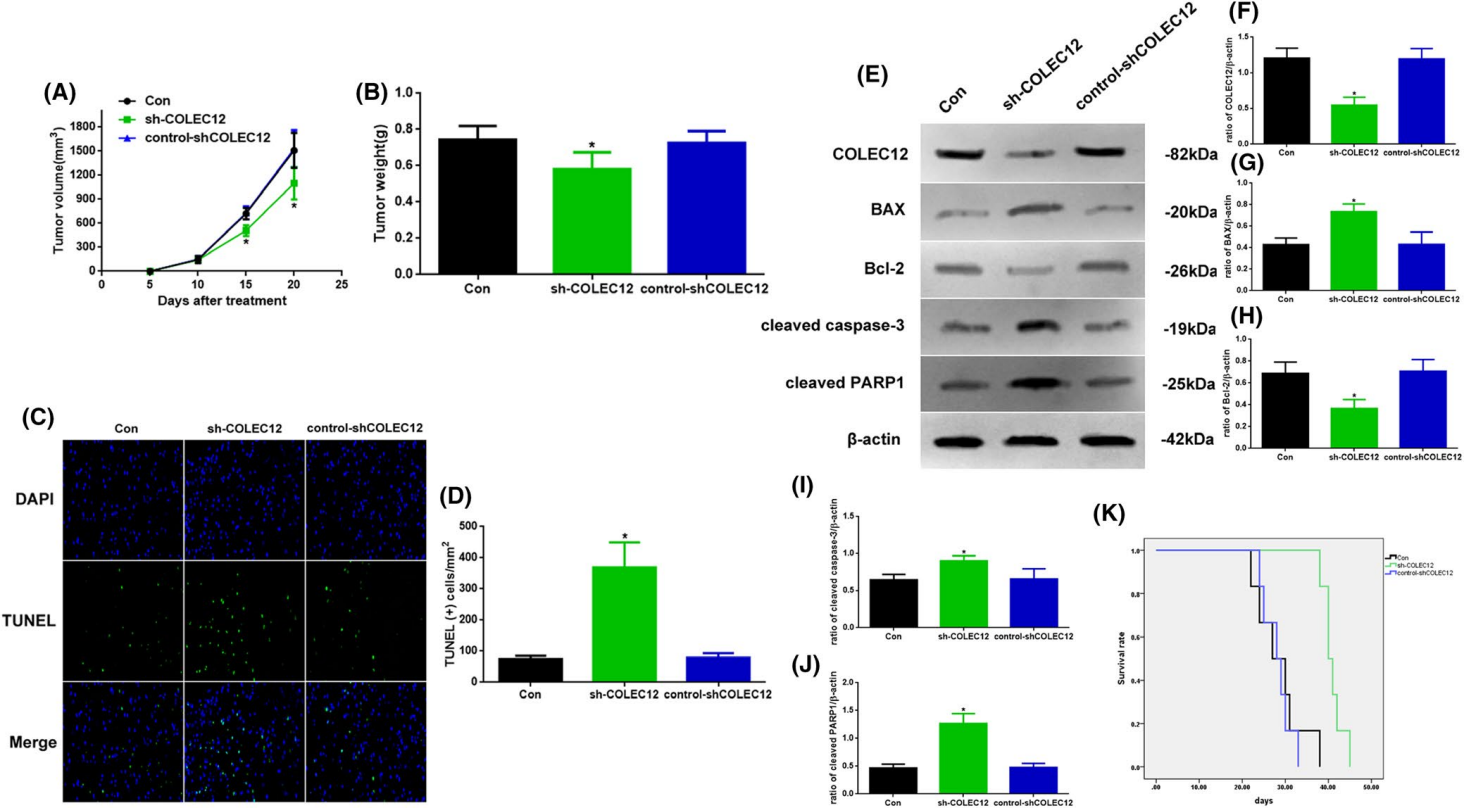
A, Tumor volume in 20‐day period and B, tumor weight at 20 days of Con, sh‐COLEC12, and control‐shCOLEC12 mouses. C, Immunofluorescence assay of TUNEL (×400) and D, TUNEL (+) cell assay. E, Western blot assay of COLEC12, BAX, Bcl‐2, cleaved caspase‐3, and cleaved PARP1 expression in vivo. Quantification of (F) COLEC12, (G) BAX, (H) Bcl‐2, (I) cleaved caspase‐3, and J, cleaved PARP1. K, Survival analysis of Con, sh‐COLEC12, and control‐shCOLEC12 mouses. Protein levels were normalized to β‐actin. (sh‐COLEC12 vs. other groups, ^*^ *P* < .05, n = 6 per group)

As COLEC12 knockdown administration could regulate osteosarcoma development in vitro, we also detected this progress in vivo (Figure 4). Firstly, we tested the tumor volume and tumor weight of the Con, sh‐COLEC12, and control‐shCOLEC12 mice. The tumor volume at 15 and 20 days in the sh‐COLEC12 mice was significantly lower than the other two groups (Figure 4A, *P* < .05). The tumor weight in sh‐COLEC12 mice was also reduced (Figure 4B, *P* < .05). Secondly, to investigate the function of apoptosis by COLEC12, we used TUNEL staining to observe the changes (Figure 4C), and the apoptotic level in sh‐COLEC12 mice was increased (Figure 4D, *P* < .05). In addition, we also detected related proteins of apoptosis by Western blot (Figure 4E). The expression of BAX, cleaved caspase‐3, and cleaved PARP1 in the sh‐COLEC12 group was increased (*P* < .05); compared with other groups, COLEC12 and Bcl‐2 showed contrary results (Figure 3F‐J). At last, the survival rate in the sh‐COLEC12 mice was significantly increased (Figure 4K, *P* < .05).

### 3.5 Knockdown of COLEC12 could increase osteosarcoma inflammation

**Figure 5:**
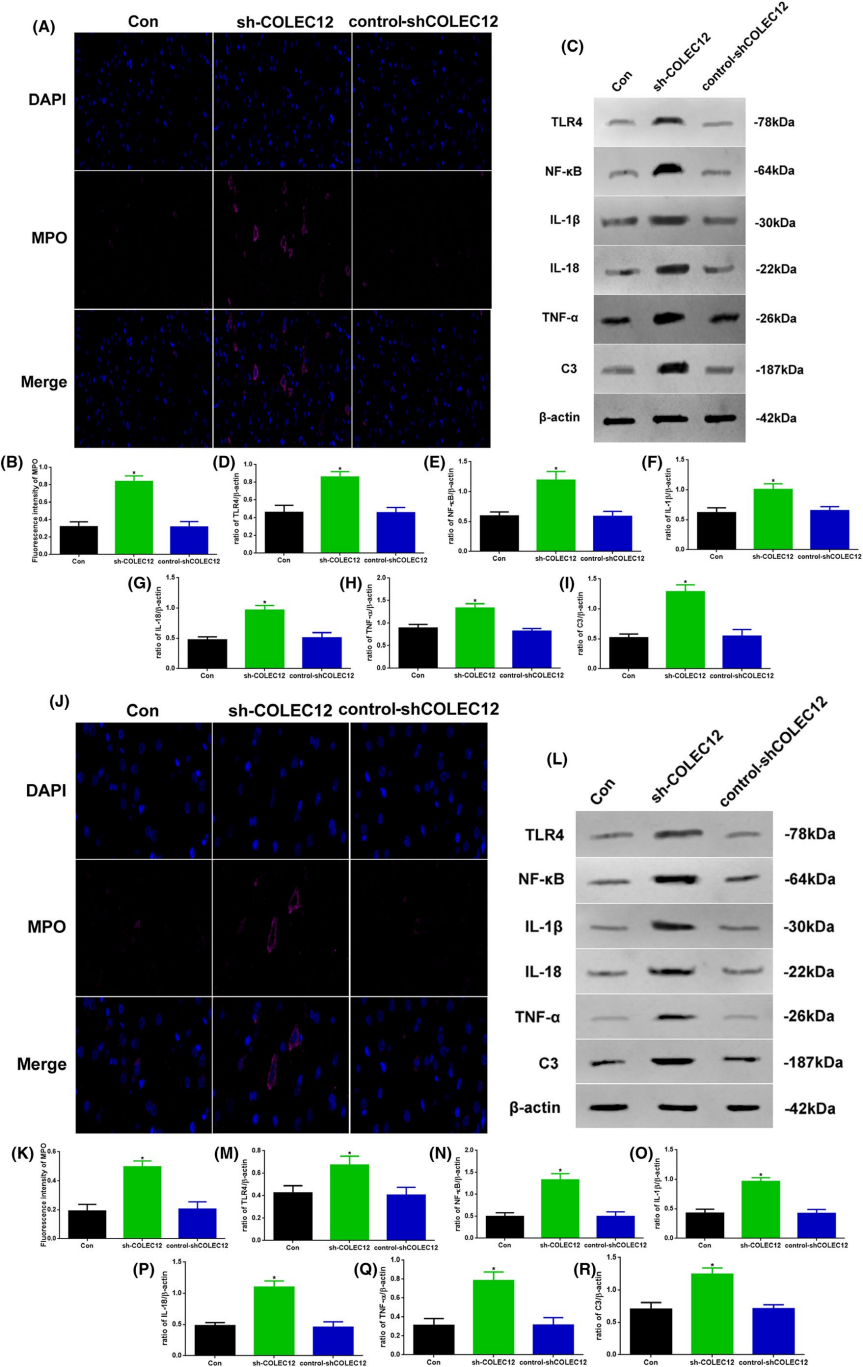
A, Immunofluorescence assay of MPO (×400) and B, fluorescence intensity assay in Con, sh‐COLEC12, and control‐shCOLEC12 mouses. C, Western blot assay of TLR4, NF‐κB, IL‐1β, IL‐18, TNF‐α, and C3 expression in vivo. Quantification of D, TLR4, E, NF‐κB, F, IL‐1β, G, IL‐18, H, TNF‐α, and I, C3. J, Immunofluorescence assay of MPO (×400) and K, fluorescence intensity assay in Con, sh‐COLEC12, and control‐shCOLEC12 mouses. L, Western blot assay of TLR4, NF‐κB, IL‐1β, IL‐18, TNF‐α, and C3 expression in vivo. Quantification of (M) TLR4, (N) NF‐κB, (O) IL‐1β, (P) IL‐18, (Q) TNF‐α, and (R) C3. Protein levels were normalized to β‐actin. (sh‐COLEC12 vs. other groups, ^*^ *P* < .05, n = 6 per group)

To further confirm whether the tumor‐regulated effect of COLEC12 is mediated through inflammation, we also observed inflammation‐related proteins by Western blot and immunofluorescence (Figure 5). In vivo, during the immunofluorescence staining of MPO (Figure 5A), the fluorescence intensity in the sh‐COLEC12 group was significantly increased (Figure 5B). As shown in Figure 5C, COLEC12 knockdown lentivirus boosted the TLR4, NF‐κB, IL‐1β, IL‐18, TNF‐α, and C3 protein levels. Compared to the other groups, the TLR4, NF‐κB, IL‐1β, IL‐18, TNF‐α, and C3 levels in the sh‐COLEC12 group were increased (Figure 5D‐I). In vitro, during the MPO immunofluorescence staining (Figure 4J), the fluorescence intensity was also significantly increased (Figure 4K). Treatment with COLEC12 knockdown lentivirus could also significantly increase TLR4, NF‐κB, IL‐1β, IL‐18, TNF‐α, and C3 level (Figure 4L‐R), which was consistent with the results in vivo.

### 3.6 The regulation of inflammation by COLEC12 in osteosarcoma was mediated thought TLR4

**Figure 6:**
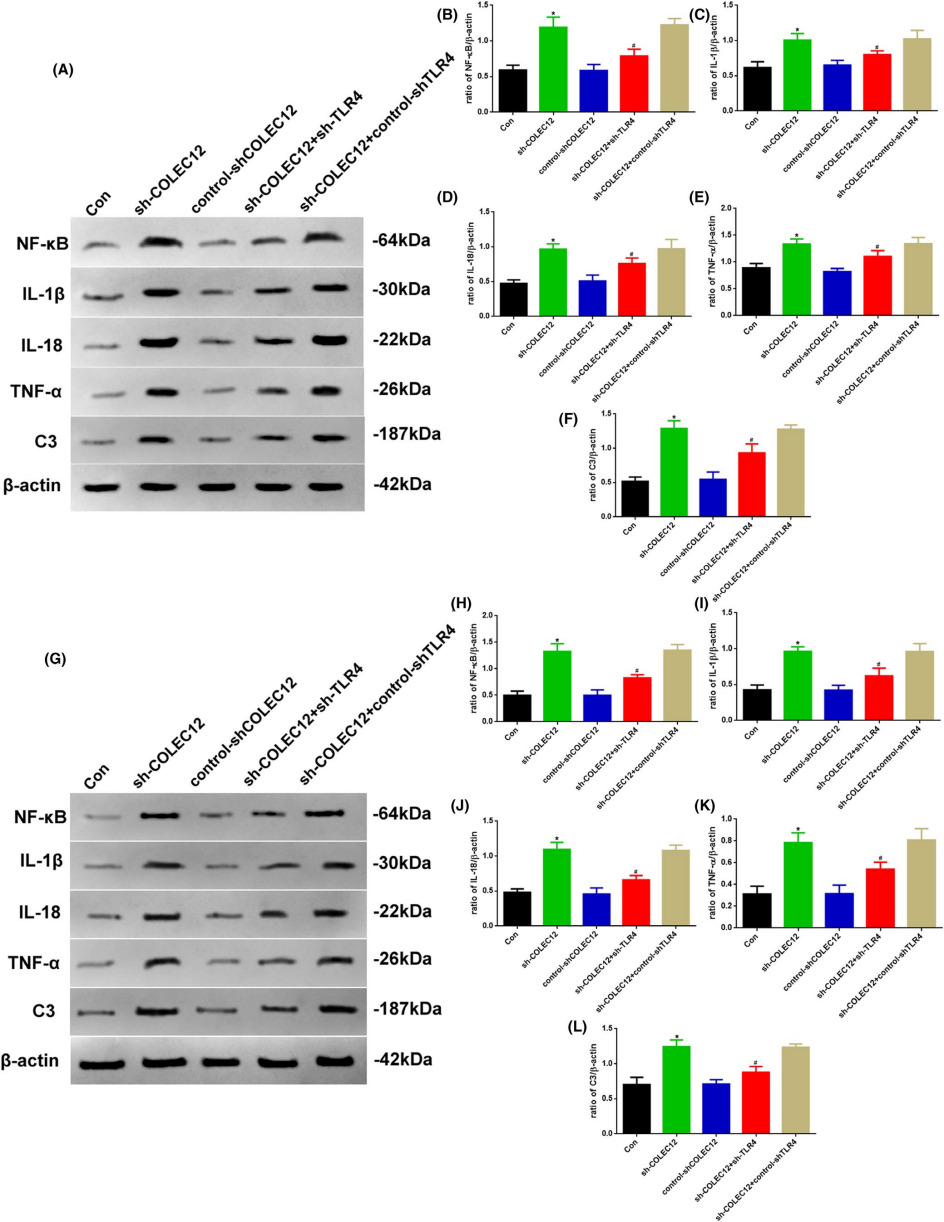
A, Western blot assay of NF‐κB, IL‐1β, IL‐18, TNF‐α, and C3 expression in Con, sh‐COLEC12, control‐shCOLEC12, sh‐COLEC12 + sh‐TLR4, and sh‐COLEC12 + control‐shTLR4 groups in vivo. Quantification of (B) NF‐κB, (C) IL‐1β, (D) IL‐18, (E) TNF‐α, and (F) C3. G, Western blot assay of NF‐κB, IL‐1β, IL‐18, TNF‐α, and C3 expression in Con, sh‐COLEC12, control‐shCOLEC12, sh‐COLEC12 + sh‐TLR4, and sh‐COLEC12 + control‐shTLR4 groups in vitro. Quantification of (H) NF‐κB, (I) IL‐1β, (J) IL‐18, (K) TNF‐α, and (L) C3. Protein levels were normalized to β‐actin. (sh‐COLEC12 vs. Con or control‐shCOLEC12 group, ^*^ *P* < .05; sh‐COLEC12 + sh‐TLR4 vs. sh‐COLEC12 or sh‐COLEC12 + control‐shTLR4 group, ^#^ *P* < .05, n = 6 per group)

Given the probable relationship between COLEC12 and TLR4 by pairwise gene correlation analysis, shown in Figure 1H, we eliminated whether TLR4 involved in COLEC12 function of osteosarcoma. In our experiment, TLR4 knockdown lentivirus was used to block TLR4 expression, in vivo and in vitro (Figure 6). As shown in Figure 6A, compared with the sh‐COLEC12 group, the NF‐κB, IL‐1β, IL‐18, TNF‐α, and C3 levels in the sh‐COLEC12 + sh‐TLR4 group were decreased in vivo (Figure 6B‐F), which indicated the regulation of inflammation by COLEC12 was possibly mediated by TLR4. The results in vitro showed similar outcomes (Figure 6G‐L).

## 4 DISCUSSION

Osteosarcoma, affecting adolescents and young adults, is one of the most common malignant bone tumors whose 5‐year overall survival (OS) rate has been 50%‐70%.^23^ Osteosarcoma has significant heterogeneity because of its histological heterogeneity and instability of its genetic makeup, which lead to its poor outcomes.^24^ Accordingly, it is warranted to find novel prognostic parameters to help improve the survival of osteosarcoma patients.

**Figure 7:**
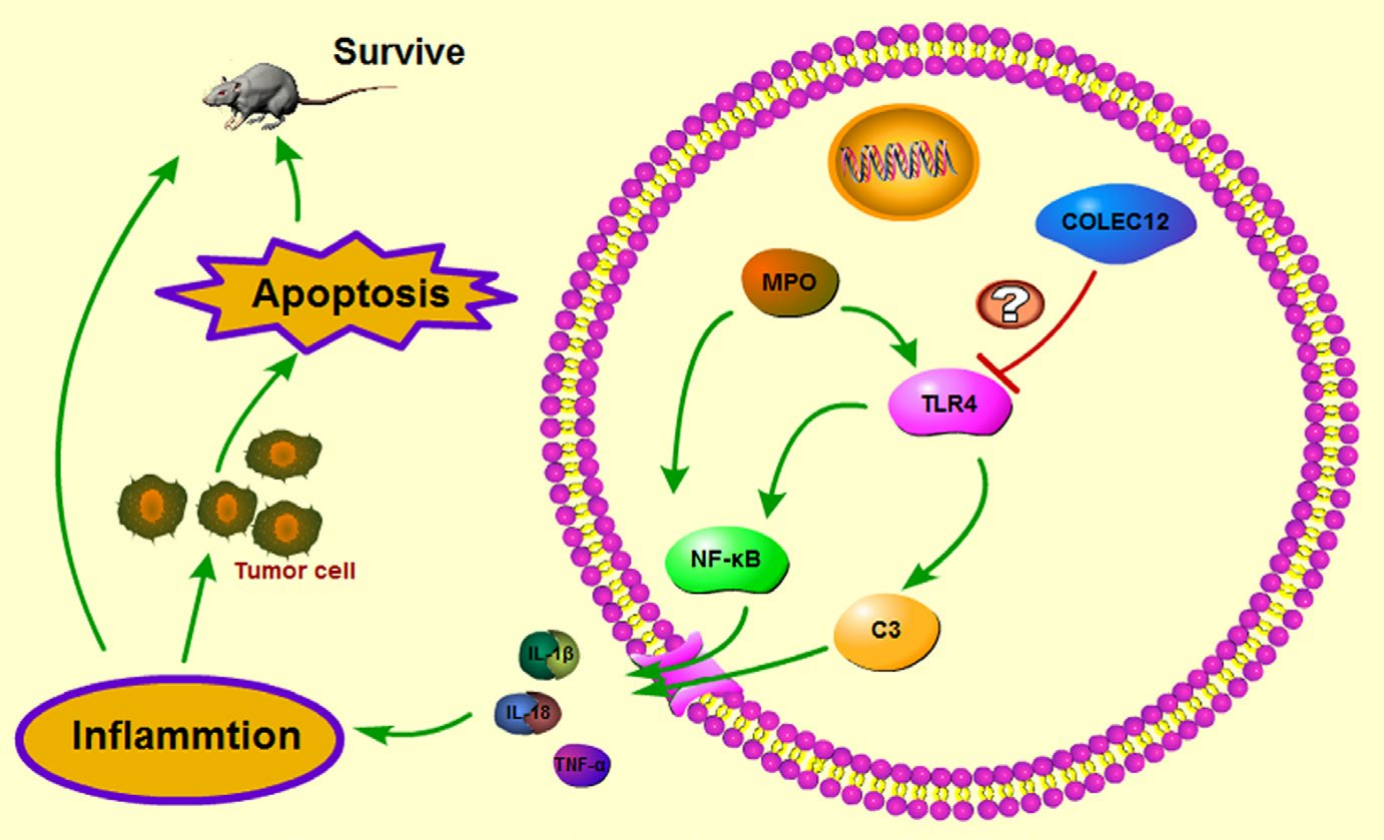
The potential mechanisms of inflammation by COLEC12 mediated through TLR4 in osteosarcoma. COLEC12 could reduce TLR4 expression, weaken NF‐κB and C3 signaling pathway to release inflammatory factors downstream, restrain immune defense to tumor cells, inhibit apoptosis of tumor cells, and cut down lives of mouses

Gene therapy is the most promising treatment and has long fascinated scientists, clinicians, and the public because of its potential to treat cancer at its genetic roots.^25^ According to the poor outcomes of osteosarcoma, searching for genes which may play a regulatory role in osteosarcoma, will guide treatment. Recently, human defense collagens, collectin‐12 (collectin placenta 1, CL‐P1, or CL‐12) derived from the COLEC12 genes, respectively, were identified and characterized.^26^ In our study, we used GEPIA and TCGA systems to predict COLEC12’s correlation in sarcoma to osteosarcoma. Results showed COLEC12 expression in SARC tumor tissue was higher than normal; high expression of COLEC12 in SARC patients had a worse prognostic outcome. In addition, a potential relationship between COLEC12 and TLR4 was revealed by pairwise gene correlation analysis. These results explained that high levels of COLEC12 may cause the development of osteosarcoma, and the role of COLEC12 may be mediated through TRL4 (Figure 7).

To verify the bioinformatic analysis of COLEC12, we detected the protein and mRNA levels of COLEC12 in different tissues and cell lines. We found the COLEC12 expression and mRNA level in tumor or Saos‐2 cells were increased which suggested COLEC12 may be related to the development of osteosarcoma. Then, in order to further investigate the function of COLEC12 in the development of osteosarcoma, we used COLEC12 knockdown lentivirus to reduce COLEC12 expression. Our results showed the inhibition of osteosarcoma development caused by COLEC12 knockdown through reducing tumor volume and weight, weakening tumor proliferation, migration, and invasion, and enhancing apoptosis. These results explain that low levels of COLEC12 could improve osteosarcoma outcomes, but the mechanism of COLEC12 is still unclear.

COLEC12 is suggested to be involved in leukocyte recruitment and cancer metastasis, and the increasing levels of COLEC12 expression were seen in cancer‐associated stromal cells,^15^, ^16^ which suggests a correlation with tumor inflammation. However, the mechanism of COLEC12 in inflammation of osteosarcoma is not fully clarified. To investigate the inflammatory function of COLEC12, we detected MPO, NF‐κB, IL‐1β, IL‐18, TNF‐α, and C3 levels, which were activated by TLR4. Our findings show that immunofluorescence staining of MPO and TLR4, NF‐κB, IL‐1β, IL‐18, TNF‐α, and C3 protein levels was increased by COLEC12 knockdown. MPO activity is always regarded as a reflection of neutrophil infiltration, which is related to TLR4/NF‐κB signaling pathway.^27^ In our study, the increasing level of MPO, complement C3, and inflammatory factors by COLEC12 knockdown could further describe the immunomodulatory function of COLEC12.

TLR4 activates overlapping signaling pathways, yet has distinct sets of target genes from other membrane receptors.^28^ Exploration of the structural details of TLR4 revealed the existence of an extracellular leucine‐rich repeat (LRR) domain, a transmembrane domain, and an intracellular Toll‐interleukin (IL)‐1 receptor (TIR) domain.^29^ TLR4 is one of the major TLRs known to play a key role in the induction of inflammatory response. Several lines of evidence have suggested a positive link between chronic inflammation and predisposition to various cancer types.^30^

TLR4 was pivotal promoter of inflammation which related NF‐kB and C3 signaling, and then released TNF‐α and ILs, which can induce apoptosis of osteosarcoma cells.^10^, ^11^, ^31^, ^32^ However, whether the role of COLEC12 for inflammation in osteosarcoma is regulated by TLR4 has not been reported. To eliminate whether TLR4 is involved in the COLEC12 inflammatory function of osteosarcoma, we used TLR4 knockdown lentivirus to block TLR4 expression, which was the preliminary component of inflammation. In our experiment, the NF‐κB, IL‐1β, IL‐18, TNF‐α, and C3 levels were decreased by TLR4 blocking. These results suggest the regulation of inflammation by COLEC12 is possibly mediated by TLR4.

Our findings indicate that COLEC12 knockdown significantly activates inflammation function and inhibits the development of osteosarcoma modulating through TLR4, and then improve the level of osteosarcoma apoptosis, prolonging the lives of mice. Taken together, these findings may provide a new strategy to osteosarcoma development by regulating inflammation.

## CONFLICT OF INTEREST

None.
